# Quantification of CD44, Epidermal Growth Factor Receptor, E-cadherin, and Vimentin in Oral Squamous Cell Carcinoma as an Indicator for Disease Progression and Survival

**DOI:** 10.7759/cureus.63000

**Published:** 2024-06-23

**Authors:** Shradha G Jaiswal, Minal Choudhary, Madhuri Gawande, Amol R Gadbail, Alka Hande, Gagan Jaiswal

**Affiliations:** 1 Oral Pathology and Microbiology, Sri Aurobindo College of Dentistry, Indore, IND; 2 Oral Pathology and Microbiology, Sharad Pawar Dental College, Datta Meghe Institute of Medical Sciences, Wardha, IND; 3 Dentistry, Indira Gandhi Medical College and Hospital, Nagpur, IND; 4 Periodontics, College of Dental Science and Hospital, Indore, IND

**Keywords:** receiver operating charecteristics, oral squamous cell carcinoma, survival, recurrence, immunohistochemistry

## Abstract

Background: Oral squamous cell carcinoma (OSCC) is commonly associated with early recurrence due to loco-regional spread. Changes at the cellular levels can be studied and are often an early indicator of disease progression, much before clinical symptoms become visible. Identifying parameters indicating an impending recurrence could help the clinician plan for early treatment and thus improve survival. Hence, this study aimed to determine if quantifiable parameters could be established for CD44, epidermal growth factor receptor (EGFR), E-cadherin, and vimentin and if these values could be used as indicators of disease progression on follow-up.

Method: A total of 150 cases of OSCC were included in the study and followed up linearly for 36 months. Paraffin-embedded tissues of these cases were subjected to immunohistochemical analysis for reactivity to CD44, EGFR, E-cadherin, and vimentin. The immunohistochemical staining correlated with the tumor's clinical and histological grade. Statistical analysis was done using SPSS Statistics version 17 (IBM Corp., Armonk, NY, USA). The receiver operating characteristics (ROC) were deployed for determining the correlation of recurrence with the immunohistochemistry (IHC) markers, while the Kaplan-Meier curve was employed for survival analysis.

Results: A recurrence rate of 70.0% and a survival rate of 66.6% were noted after a follow-up period of three years. It was found that both CD44 and E-cadherin decreased with the grade of tumor, while EGFR and vimentin increased with tumor de-differentiation. The E-cadherin was found to be the best predictor of recurrence and survival among all the four markers.

Conclusion: The cut-off values could be identified for all four biomarkers, which on follow-up proved to be a valuable tool with a high sensitivity and specificity for predicting recurrence and three-year survival in patients with OSCC.

## Introduction

The behavior of oral squamous cell carcinoma (OSCC) is quite unpredictable concerning treatment and prognosis, due to its high propensity for locoregional metastasis. Psychological and social stigma associated with oral lesions is a bigger problem than the disease itself, as the routinely employed therapeutic protocol results in decreased quality of life. If changes at the cellular level are studied, they may prove to be valuable in improvising treatment protocols without actually compromising living standards. Due to this, more focus is laid on markers that can identify subtle changes at the cellular level, which can be used to predict tumor aggressiveness. Markers of cell proliferation, angiogenesis, cell adhesion, epithelial-mesenchymal transition, and oncogenes have been extensively studied as tools that can be used to predict patient prognosis. Deploying some of these markers at the time of treatment planning can be used to customize treatment protocols to make them tailor-made for particular patients.

The CD44 is a cell adhesion molecule that attaches to ligands in the connective tissue like hyaluronic acid, osteopontin, and matrix metalloproteinase, thereby increasing the degradation of cell-matrix junctions, which is an important event for the tumor cells to invade the connective tissue [1]. The E-cadherin is an 'adhesion molecule' responsible for the attachment of one cell to another. Its expression changes with alterations in the architectural morphology, as seen in dysplasia and malignancy. Downregulation of these cell adhesion molecules increases the capacity of these modified cells to disengage from each other, connective tissue, and the extracellular matrix, thereby increasing cell migration [2]. Keeping this in mind, we realized that understanding the expression of both CD44 and E-cadherin together is essential to understanding the basic pattern of cell motility and migration. Epidermal growth factor receptor (EGFR) is a marker of cell cycle acceleration and proliferation, and its expression is increased in aggressive malignancies of the head and neck [3]. Vimentin is a mesenchymal marker but has been proven to play an important role in epithelial-mesenchymal interactions [4].

After reviewing the available literature, we shortlisted these four biomarkers as they have varied mechanisms of action and appear to be of great value in the process of carcinogenesis. These biomarkers would help us better characterize these tumors based on their biological aggressiveness and predict treatment outcomes. Hence, we aimed to evaluate if quantifiable parameters could be established for the expression of CD44, EGFR, vimentin, and E-cadherin in terms of predicting tumor aggressiveness and prognostic significance, if any, in OSCC.

## Materials and methods

This research was carried out at the Department of Oral and Maxillofacial Pathology and Microbiology, Sharad Pawar Dental College and Hospital, Datta Meghe Institute of Medical Sciences (Wardha, Maharashtra, India). The nature of the study is an ambispective cohort, as cases registered in the past three years were included in the study and followed linearly for a period of 36 months, with the last case being followed up to December 2016. This study used paraffin-embedded, formalin-fixed tissue samples from the archives of the department. Ethical clearance was duly taken from the Institutional Ethics Committee (approval no. DMIMS (DU)/IEC/2015-16/1578).

Inclusion and exclusion criteria

Histopathologically confirmed cases of oral cancer, which received only surgical debulking of the tumor as treatment, were included. Recurrence and survival at the end of three years (36 months) were also taken into account. The sample size was determined using the criteria given by Krejcie et al. [5]. The total sample included in this study comprised 150 patients with three-year survival data. Samples were divided into three groups, namely group A with 50 cases of well-differentiated squamous cell carcinoma (WDSCC), group B with 50 cases of moderately differentiated squamous cell carcinoma (MDSCC), and group C with 50 cases of poorly differentiated squamous cell carcinoma (PDSCC).

Cases of recurrence, patients undergoing chemotherapy or radiotherapy, and patients with any coexisting malignancy were excluded from the study. Keeping the sample size in mind, 200 cases were included in the initial, potentially eligible phase. Patients in which some part of the data couldn’t be accessed, such as clinical staging, patients who refused to participate in the study, or those who couldn’t be contacted due to any reason, etc., were excluded from the study. Thus, a total of 176 cases were included in the study, of which 15 cases were lost to follow-up. From the remaining 161 cases that were followed up, 150 were included for the ease of statistical analysis so that an equal number of cases could be examined in each group.

Immunohistochemistry procedure

**Figure 1:**
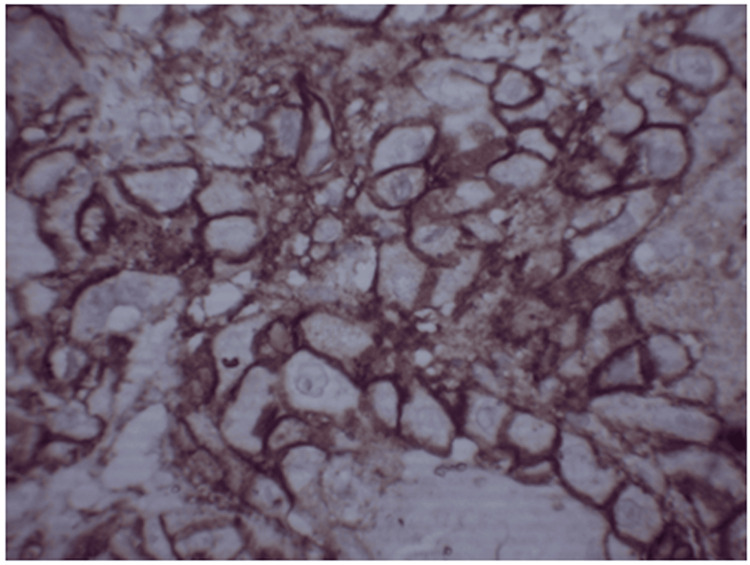
Photomicrograph showing CD44 expression in well-differentiated OSCC at 400x OSCC: Oral squamous cell carcinoma

**Figure 2:**
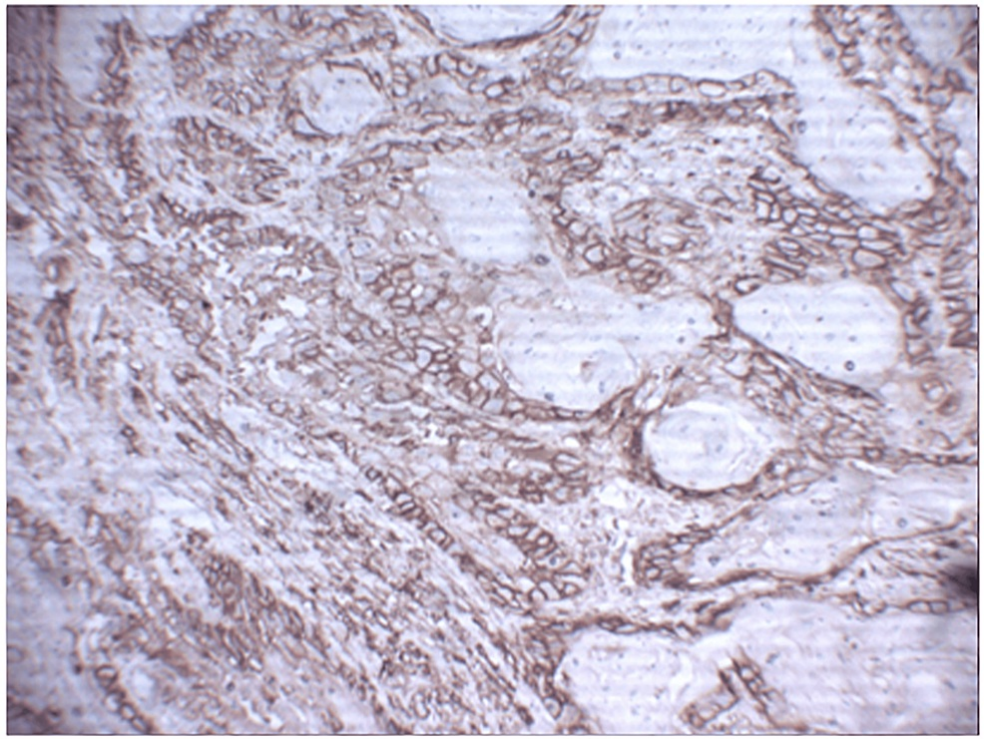
Photomicrograph showing E-cadherin expression in well-differentiated OSCC at 400x OSCC: Oral squamous cell carcinoma

**Figure 3:**
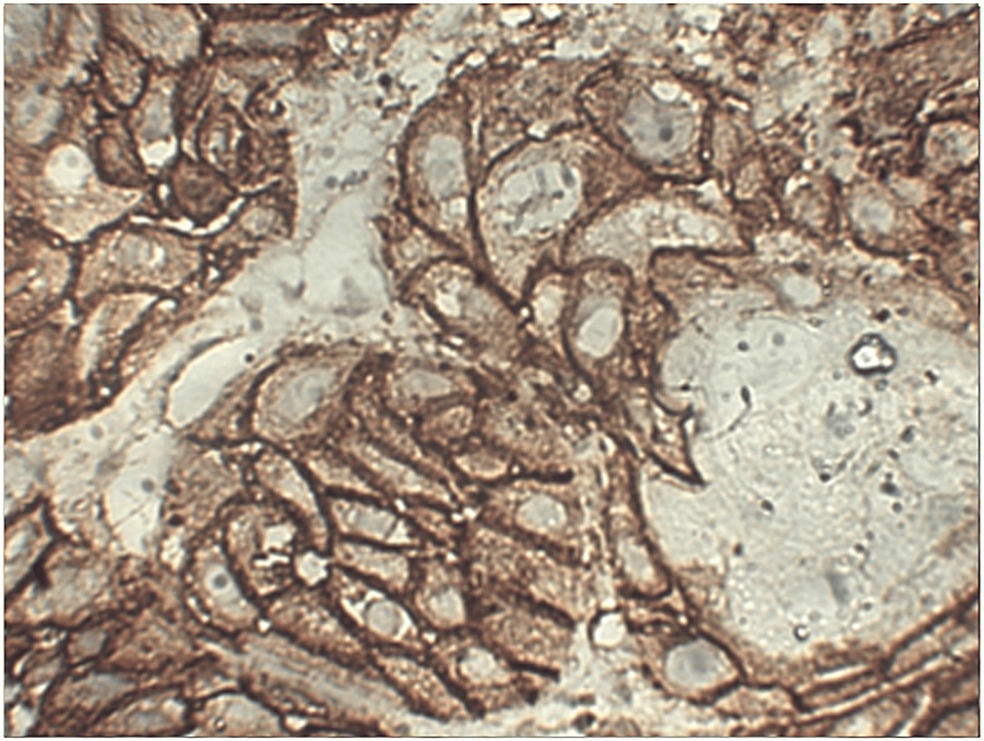
Photomicrograph showing EGFR expression in well-differentiated OSCC at 400x EGFR: Epidermal growth factor receptor, OSCC: Oral squamous cell carcinoma

**Figure 4:**
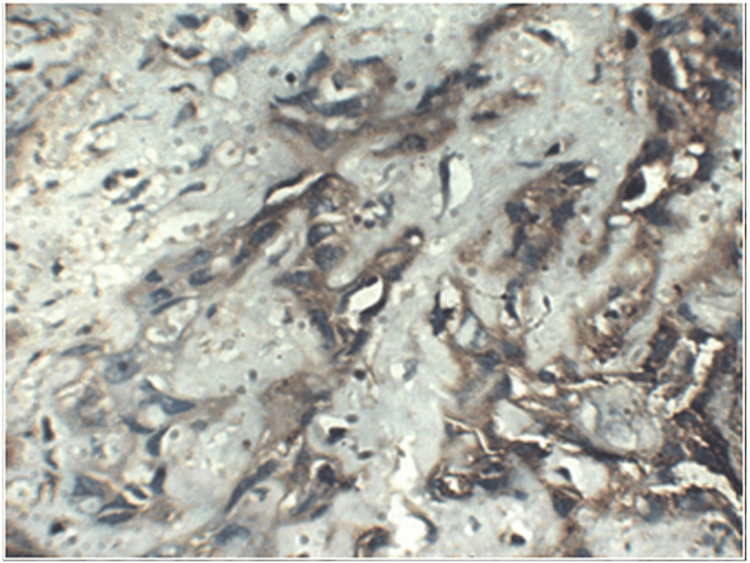
Photomicrograph showing vimentin expression in poorly differentiated OSCC at 400x OSCC: Oral squamous cell carcinoma

The staining was carried out on tissues fixed in formalin, which had its pH neutralized to alkaline property to detect expression of the antigens. The standard immunohistochemistry procedure was carried out for expression of CD44, EGFR, E-cadherin, and vimentin (Figures 1-4). Antibodies employed were CD44 antibody (mouse monoclonal, clone: 56-3C11; Lab Vision Corp., Fremont, CA, USA), pre-diluted EGFR antibody (EGFR, clone EP38Y; Lab Vision Corp.), E-cadherin antibody (ready to use pre-diluted, mouse monoclonal; Lab Vision Corp.), and vimentin antibody (pre-diluted mouse monoclonal; Lab Vision Corp.). Histological grading was performed by two oral pathologists who independently graded the OSCC cases using Broder’s grading system and were blinded to the clinical grading and staging of patients and also to the findings of each other.

Scoring of immunohistochemical staining

Stained slides were visualized for CD44, EGFR, E-cadherin, and vimentin staining using the Leica DM LB2 microscope (Leica Microsystems, Wetzlar, DEU) at low power (100x) followed by high power (400x) magnification. The presence and location of brown color were accepted as a positive staining reaction. The absence of this staining in negative control was employed as a marker for the specificity of the antibody. The scoring criteria for CD44 were deployed as laid out by Hema et al. [6]. The degree of positive staining for CD44 antibody was evaluated using a semi-quantitative scoring system on a scale of 1 to 4 for intensity (I): 1 = none, 2 = mild, 3 = moderate, and 4 = strong. For Distribution (D), the scoring was 1 = none, 2 = focal, 3 = patchy, and 4 = diffuse. Tissues with I×D ≤4 were considered weakly positive (for example, I = 2 x D = 2 will give a total score of 4 and hence would be considered weakly positive), and those with I×D >4 were designated strongly positive. For example, I = 3 x D = 2 will give a total score of 6 and hence would be considered strongly positive.

Scoring for EGFR was done using the criteria laid out by Sarkis et al. [7]. The extent of EGFR immunostaining was graded and scored as follows: 0 points for negative staining of the considered cells, 1 point for <10%, 2 points for 10% to 50%, 3 points for 51% to 80%, and 4 points for ≥ 80% positive staining of the considered cells. Scoring for E-cadherin and vimentin was measured using a numerical scale (-: negative expression; +: weak expression; ++: moderate expression; and +++: strong expression). The percentage of cells stained at each intensity level was graded as 0 (<5%), 1 (5% to 25%), 2 (26% to 50%), 3 (51% to 75%), and 4 (>75%). The intensity score and percentage of positive cells were multiplied to derive the final scores. The evaluation of all the markers was carried out at the tumor's front region.

Statistical analysis

The obtained data was analyzed using SPSS Statistics version 17.0 (IBM Corp., Armonk, NY, USA). Comparison of expressions of CD44, EGFR, vimentin, and E-cadherin was carried out amongst histopathological grades and recurrent and non-recurrent OSCC by using one-way ANOVA along with post hoc Tukey’s honestly significant difference (HSD) and Student's t-test, respectively. The receiver-operating characteristic (ROC) curve was plotted employing recurrence as the dependent variable and markers (CD44, EGFR, E-cadherin, and vimentin) as independent variables. The cut-off points were identified by selecting the optimal sensitivity with specificity. Kaplain-Meier survival analysis was performed to identify the correlation between three-year survival and expression of CD44, EGFR, E-cadherin, and vimentin. The probability value from p<0.01 to p<0.001 was considered statistically significant.

## Results

Out of a population of 150, the age of all cases (n = 150) with OSCC was found to be in the range of 35 to 69 years with a mean (mean ± standard deviation) age of 53.74±10.51 years (Table 1).

**Table 1:** Frequency and percentage distribution of age among patients in the three groups The data is represented as number (n) and percentage (%).

Age (years)	Group A		Group B		Group C	
	n	%	n	%	n	%
35-44	10	20.0	8	16.0	7	14.0
44-53	18	36.0	13	26.0	21	42.0
53-62	12	24.0	14	28.0	11	22.0
62-71	10	20.0	15	30.0	11	22.0
Total	50	100.0	50	100.0	50	100.0

Buccal mucosa was the most common site of lesion (76.0%, n = 114) followed by gingiva (32.2%, n = 8), while the tongue was the least common site of biopsy (4.4%, n = 8) as shown in Table 2. 

**Table 2:** Assessment and comparison of biopsy site among OSCC patients The data is represented as number (n) and percentage (%). OSCC: Oral squamous cell carcinoma

Site of lesion	Group A		Group B		Group C	
	n	%	n	%	n	%
Buccal mucosa	41	82.0	39	78.0	34	68.0
Gingiva	8	16.0	10	20.0	10	20.0
Tongue	1	2.0	1	2.0	6	12.0
Total	50	100.0	50	100.0	50	100.0

Around 20% of WDSCC (n = 10) showed metastasis of regional nodes, followed by MDSCC at 22.0% (n = 11) and PDSCC at 68.0% (n = 29). Metastasis in a single ipsilateral lymph node (≤ 3 cm) (N1), was seen in 20% (n = 10) of WDSCC cases, 22.0% (n = 11) of PDSCC cases, and 18.0% (n = 9) of MDSCC. In group 2, metastasis in a single ipsilateral lymph node measuring more than 3 cm but not more than 6 cm in greatest dimension; or in multiple ipsilateral lymph nodes, none more than 6 cm in greatest dimension; or in bilateral or contralateral lymph nodes, none greater than 6 cm in greatest dimension (N2) was seen in 36.0% (n = 18) of PDSCC and 4.0% (n = 2) of MDSCC. The well-differentiated cases did not show N2 metastasis (Table 3). 

**Table 3:** Assessment and comparison of regional lymph nodes among OSCC patients The data is represented as number (n) and percentage (%). OSCC: Oral squamous cell carcinoma

Regional lymph nodes	Well-differentiated		Moderately differentiated		Poorly differentiated	
	n	%	n	%	n	%
No regional node metastasis	40	80.0	39	78.0	21	42.0
N1	10	20.0	9	18.0	11	22.0
N2	0	0.0	2	4.0	18	36.0
Total	50	100.0	50	100.0	50	100.0

The mean size of the tumor in PDSCC patients (4.73 cm) was found to be significantly greater compared to that of MDSCC (3.97 cm) and WDSCC (2.40 cm) patients. One-way ANOVA showed that the differences in mean sizes of tumors of OSCC patients were statistically strongly significant (p = 0.000) among the three grades of OSCC. Comparison in time of recurrence of OSCC indicated that the mean time of recurrence (6.32 months) in PDSCC patients was found to be significantly lesser compared to MDSCC (17.76 months) and WDSCC (36.00 months) patients. One-way ANOVA showed that the differences in the mean time of recurrence of OSCC patients were statistically strongly significant (p = 0.000) among the three grades of OSCC (Table 4). 

**Table 4:** Comparison of tumor size, time of recurrence, and tumor grade among OSCC patients The mean differences are highly significant at the 0.001 level of significance. OSCC: Oral squamous cell carcinoma, WDSCC: Well-differentiated squamous cell carcinoma, MDSCC: Moderately differentiated squamous cell carcinoma, PDSCC: Poorly differentiated squamous cell carcinoma, LOS: Level of significance

Parameter		Mean	± SD	Standard error	F-statistic	p-value (LOS)
Tumor size (cm)	WDSCC	2.40	± 0.52	0.07	108.14	p=0.000*
	MDSCC	3.97	± 1.02	0.14		
	PDSCC	4.73	± 0.81	0.11		
Time of recurrence (month)	WDSCC	36.00	± 0.00	0.00	39.78	p=0.000*
	MDSCC	17.76	± 12.54	1.77		
	PDSCC	6.32	± 12.18	1.72		

One-way ANOVA was used to identify the significance of mean differences in expressions of CD44, EGFR, vimentin, and E-cadherin and time of recurrence among diagnosed cases of WDSCC, MDSCC, and PDSCC to rule out the differences and usefulness of expressions of CD44, EGFR, vimentin, and E-cadherin as an indicator for improving the early assessment of patients indicated for OSCC. Further, the inter-group differences were evaluated using the post-hoc test using Tukey's HSD test (Table 5). 

**Table 5:** Comparison of expressions of CD44, EGFR, vimentin, and E-cadherin and the time of recurrence among the three grades of OSCC Values expressed as mean +/- SD. The mean differences are highly significant at the 0.001 level of significance. OSCC: Oral squamous cell carcinoma, EGFR: Epidermal growth factor receptor, WDSCC: Well-differentiated squamous cell carcinoma, MDSCC: Moderately differentiated squamous cell carcinoma, PDSCC: Poorly differentiated squamous cell carcinoma, LOS: Level of significance

Expression	Grades of OSCC	Mean ± SD	F-statistic	p-value (LOS)
CD44	WDSCC	12.00±0.00	256.49	p = 0.000
	MDSCC	7.80±1.70		
	PDSCC	5.92±1.66		
EGFR	WDSCC	1.00±0.0	45.29	p = 0.000
	MDSCC	1.26±0.53		
	PDSCC	2.04±0.83		
Vimentin	WDSCC	0.00±0.0	82.17	p = 0.000
	MDSCC	1.74±1.45		
	PDSCC	3.76 ± 2.09		
E-cadherin	WDSCC	9.00±0.00	195.74	p = 0.000
	MDSCC	5.44±1.20		
	PDSCC	3.56±2.10		
Time of recurrence (month)	WDSCC	35.0± 1.00	39.78	p = 0.000
	MDSCC	17.76±12.54		
	PDSCC	6.32±12.18		

**Figure 5:**
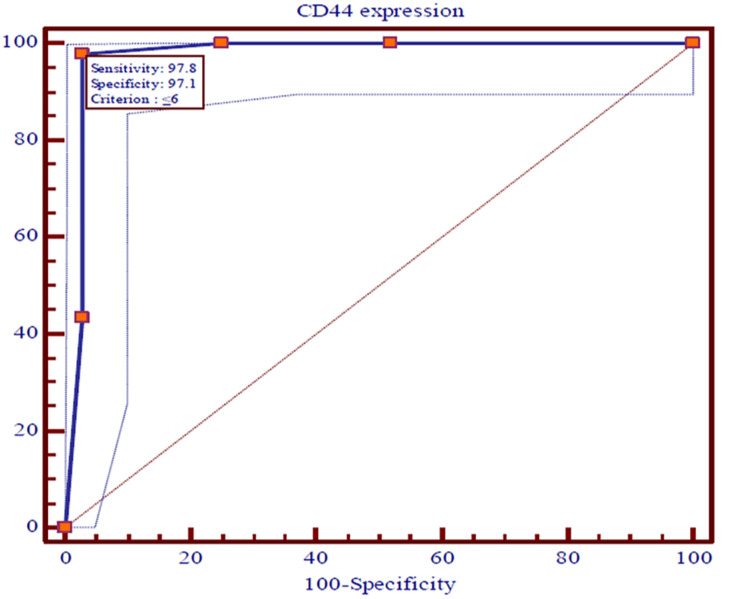
The ROC curves showing the AUC along with optimal sensitivity and specificity at the specific cut-off point for expressions of CD44 among cases of OSCC (p<0.001) ROC: Receiver operating characteristic, AUC: Area under the curve, OSCC: Oral squamous cell carcinoma

**Figure 6:**
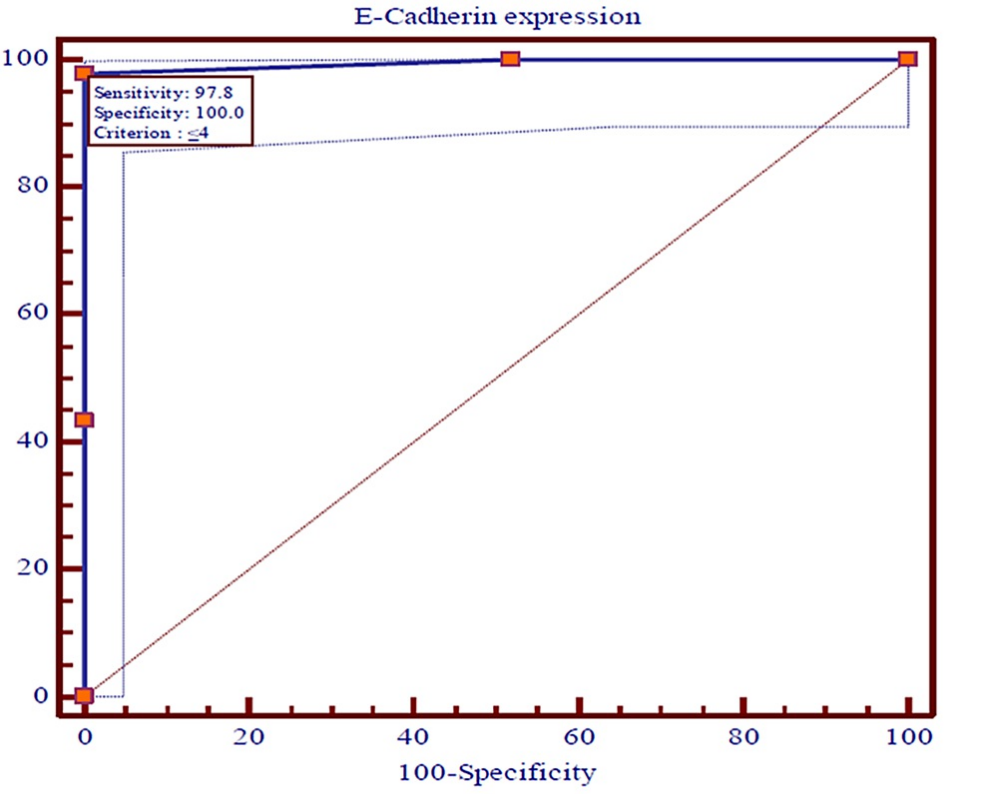
The ROC curves showing the AUC along with optimal sensitivity and specificity at the specific cut-off point for expressions of E-cadherin among cases of OSCC (p<0.001) ROC: Receiver operating characteristic, AUC: Area under the curve, OSCC: Oral squamous cell carcinoma

**Figure 7:**
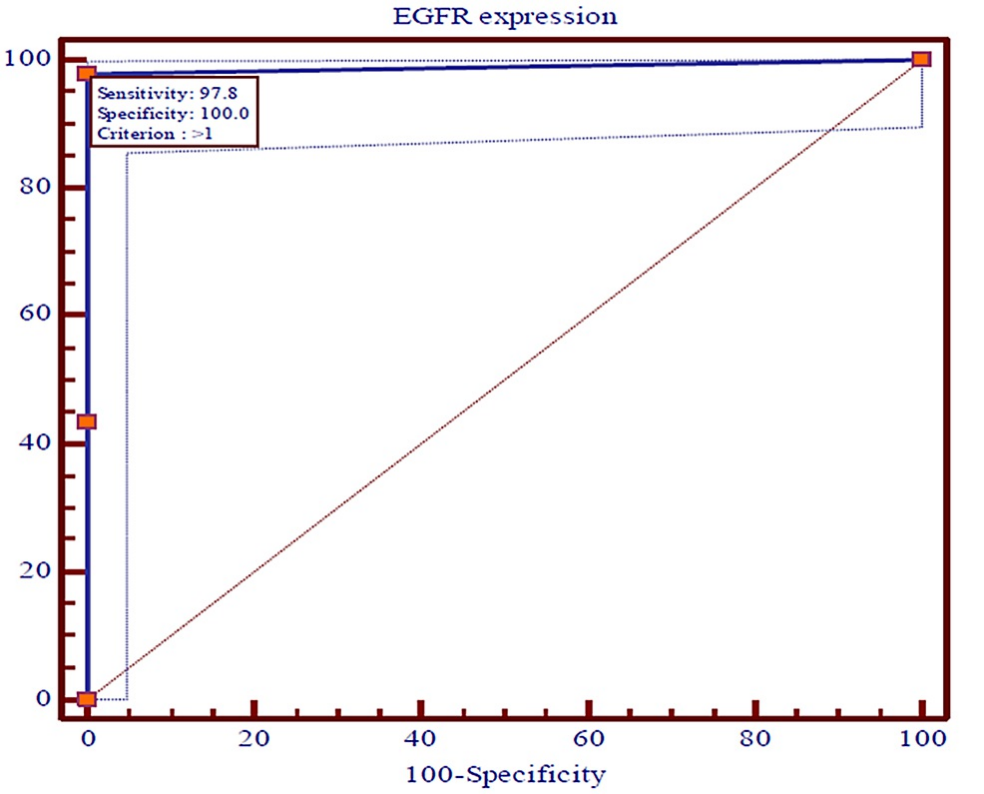
The ROC curves showing the AUC along with optimal sensitivity and specificity at the specific cut-off point for expressions of EGFR among cases of OSCC (p<0.001) ROC: Receiver operating characteristic, AUC: Area under the curve, OSCC: Oral squamous cell carcinoma, EGFR: Epidermal growth factor receptor

**Figure 8:**
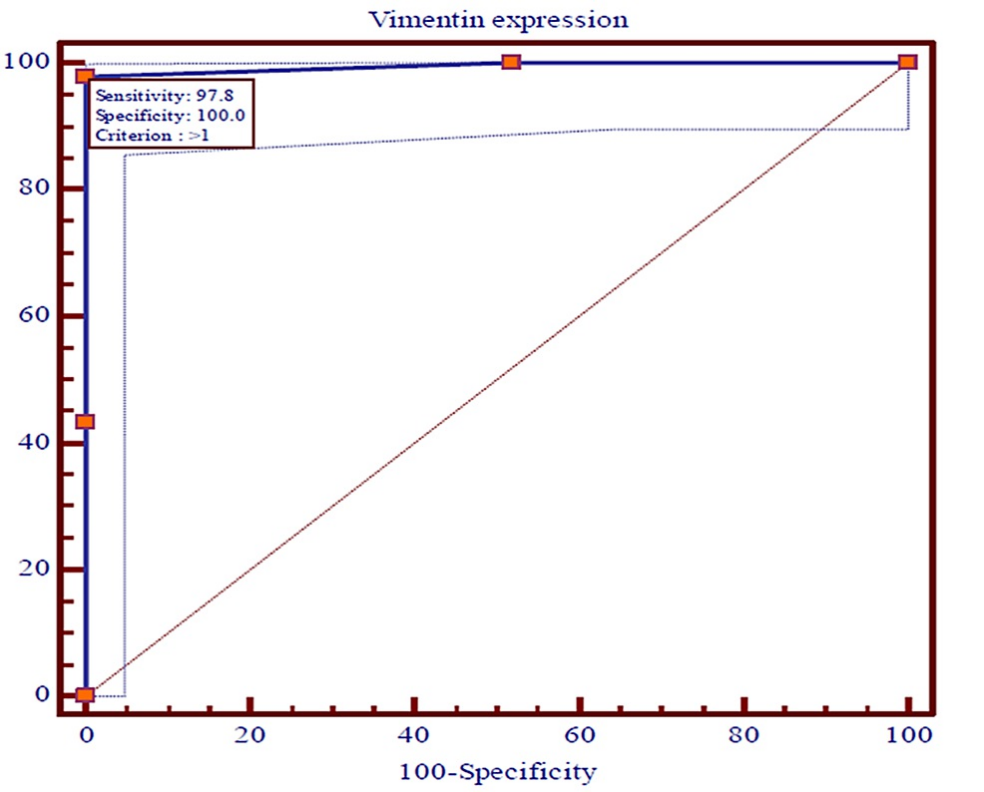
The ROC curves showing the AUC along with optimal sensitivity and specificity at the specific cut-off point for expressions of vimentin in cases of OSCC (p<0.001) ROC: Receiver operating characteristic, AUC: Area under the curve, OSCC: Oral squamous cell carcinoma

The ROC curves were used to predict the recurrences among OSCC patients using expressions of CD44, EGFR, vimentin, and E-cadherin and survival at the end of 36 months individually. The recurrence of OSCC in patients was utilized as a classification variable (dependent factor), while the expressions of CD44, EGFR, vimentin, and E-cadherin and survival at the end of 36 months acted as test variables (independent factor). The cut-off point for recurrences of OSCC was projected by selecting the optimal sensitivity with specificity of expressions of CD44, EGFR, vimentin, and E-cadherin and survival at the end of 36 months and was evaluated statistically. The expressions of CD44, EGFR, vimentin, and E-cadherin and survival at the end of 36 months were the most significant (p<0.0001) predictors of recurrences of OSCC among patients (Figures 5-8). 

The ROC curves analyze and provide a comprehensive and visually attractive way to summarize the accuracy of predictions of recurrences of OSCC with a 95% confidence interval of area under the curve (AUC) using expression of CD44, EGFR, vimentin, and E-cadherin and survival at the end of 36 months among previously diagnosed cases of OSCC. The ROC curve shows the trade-off between sensitivity and specificity and detects the performance of a developed test parameter, which classifies cases into two categories, i.e., with and without recurrences of OSCC. The closer the curve follows the left-hand border and then the top border of the ROC space, the more accurate the test parameter. Accuracy is measured by the area under the ROC curve. An area of 1 represents a perfect test, while an area of 0.5 represents a worthless test. The AUC for expressions of vimentin and E-cadherin were the maximum (0.994), while 0.989 and 0.975 were documented for expressions of EGFR and CD44, respectively. Survival among patients of OSCC for three years was found to be 0.717. All the AUCs were found to be statistically highly (p<0.0001) significant. 

The sensitivity, specificity, positive predictive value (PPV), and negative predictive value (NPV) with criteria of test variables that were observed as predictors of recurrence of OSCC are shown in Table 6. The cut-off point for recurrence of OSCC was identified by selecting the optimal sensitivity with specificity of expressions of CD44, EGFR, vimentin, and E-cadherin and survival at the end of 36 months. The diagnostic accuracy of test variables to predict recurrence of OSCC using sensitivity, specificity, PPV, and NPV has indicated that the expressions of CD44 were noted with an optimal sensitivity of 97.83% and a specificity of 97.12%, while expressions of EGFR, vimentin, and E-cadherin were observed with similar optimal sensitivity of 97.83% and specificity of 100.0%. The optimal sensitivity (43.80%) of survival of OSCC cases at the end of 36 months was found to be weak with the maximum specificity (100.0%). The PPV of expression of CD44 was a little smaller (93.70%) than NPV (99.00%), while the PPVs of EGFR, vimentin, and E-cadherin were found to be similar and a little higher (100.00%) compared to the NPVs (99.00%). Nevertheless, the PPV of survival among cases of OSCC at the end of 36 months was the maximum (100.00%) compared to NPV (80.00%).

**Table 6:** Diagnostic performances of test variables to judge recurrences of OSCC OSCC: Oral squamous cell carcinoma, EGFR: Epidermal growth factor receptor

Test variables	Cut-off point	Sensitivity	Specificity	Positive predictive value	Negative predictive value
CD44 expression	≤6	97.83%	97.12%	93.70%	99.00%
EGFR expression	>1	97.83%	100.00%	100.00%	99.00%
Vimentin expression	>1	97.83%	100.00%	100.00%	99.00%
E-cadherin expression	≤4	97.83%	100.00%	100.00%	99.00%
Recurrence in months	≤35	43.80%	100.0%	100.00%	80.00%

## Discussion

Considerable work has been carried out in the past few years to identify the factors responsible for the aggressiveness of oral cancer. Many features have been used with varying degrees of success, ranging from the tumor, node, metastasis (TNM) classification system to histological grading. However, none of these can accurately pinpoint the pattern of behavior of tumor cells, and this has resulted in a static survival rate of about 50% for the last few years. In tumors of other parts of the body, there has been a progressive improvement in the survival rate. This dichotomy is indicative of shortcomings in our treatment protocols. Assessment of a panel of biomarkers that can segregate aggressive tumors at the time of treatment planning can be used to customize treatment protocols for particular patients.

We found that a total of 33.33% of cases showed regional nodal metastasis. On correlating nodal metastasis with tumor grade, we found that 20% of cases of well-differentiated tumors had regional nodal metastasis, followed by 42% in moderately and 58% in poorly differentiated cases of OSCC, thus indicating that lymph node metastasis increases with the increasing grade of squamous cell carcinoma (SCC). Our finding was consistent with other studies [8,9].

On correlating survival at the end of three years with the grade of the tumor, we found a survival rate of 66.6%. This is in accordance with another study [10]. We further compared survival rate with tumor differentiation, and we found that patients with well-differentiated tumors showed the highest survival of 80.0%, followed by moderately differentiated at 72.0% and poorly differentiated at 48.0% at the end of 36 months. This proves that dedifferentiation results in tumor aggressiveness and hence reduced survival. On calculating survival at the end of three years, it was found to be an average of 66.6%. On comparing the survival rate with tumor differentiation at the end of 36 months, we found it to be 80% in WDSCC, 72% in MDSCC, and 48% in PDSCC among patients with OSCC.

It was found that the immunostaining of CD44 and E-cadherin was inversely related to differentiation and decreased progressively from well to moderately to poorly differentiated SCC, thereby confirming the role of CD44 and E-cadherin as a cell adhesion molecule aiding the motility of transformed cells. This finding was found to be in correlation with other studies that found that E-cadherin expression is normal in unaffected epithelium but gets upregulated in severe dysplasias and is downregulated in carcinomas [11,12]. In studying the survival rate of OSCC cases concerning CD44 and E-cadherin, it was observed that the survival rate reduced with the decreasing levels of CD44 and E-cadherin. This relation between survival and CD44 and E-cadherin levels was found to be statistically significant.

We found that EGFR was directly related to tumor differentiation, such that its expression increased with increasing grade of the tumor, and this was found to be statistically highly significant among the three grades (p = 0.000) of OSCCs. This shows that EGFR is up-regulated with increasing differentiation, and its overexpression was associated with recurrence and poor disease-free survival. This is per other studies that show that EGFR expression was downregulated with increasing grade of tumor in OSCC [13].

It was found that vimentin was expressed by the epithelial islands only in poorly differentiated tumors. This finding was similar to the ones described by other studies [14-16]. We did not find much expression of vimentin in the epithelial islands of well- and moderately differentiated tumors; this was found contrasting to the claimed by other studies [17,18]. This shows that vimentin expression is regularly seen in the connective tissue; however, the dysplastic epithelial cells start expressing vimentin in only high-grade tumors, supporting the concept of epithelial-mesenchymal transition.

On plotting the ROC curve, we found a cut-off value of ≤ 6 for CD44; for EGFR and vimentin, the cut-off value was >, while for E-cadherin it was < 4. These values give a fair idea with the help of which immunohistochemistry (IHC) score can be utilized for predicting recurrence. So if the value of the marker falls below the cut-off value, it will be indicative of loco-regional spread, indicating a tendency for recurrence and decreased survival. So far no other study has identified this correlation.

The extent of the research gap filled is that we have been able to validate the utility of CD44, EGFR, E-Cadherin, and vimentin as indicators of tumor aggressiveness and recurrence. We have also been able to identify the cut-off values for all four biomarkers, which can be used as a baseline by clinicians for treatment planning and determining the superiority of E-cadherin in all the parameters.

The limitations of the study were that due to time constraints and this being a linear study, a follow-up of only three years was possible. Also, the computed score obtained was a semi-quantitative one. Hence, further scope of the study includes multi-centric studies on these markers to understand the utility of the computed score and follow-up studies for a period of five years for survival data to further validate the computed score generated by us for CD44, EGFR, E-cadherin, and vimentin for recurrence and prognosis.

## Conclusions

This study was designed using CD44, EGRF, E-cadherin, and vimentin, as limited information can be gathered by quantitative studies; hence, it was an attempt to generate a score using quantitative and qualitative parameters. On analysis of the three-year survival by Kaplan-Meier curve, it could be concluded that all four markers are good predictors of survival, with E-cadherin and vimentin being the best predictors of survival. Based on the results of this study, the information generated could be availed to segregate tumors into aggressive and non-aggressive variants, thereby helping design specific treatment protocols that would increase the survival of the patient. All in all, an E-cadherin value of 1 and a vimentin value of 6 will be an indicator of poor survival in patients with OSCC. The study anticipates an increase in the survival rate with the use of these parameters. Thus the translatory component achieved is that the computed score of CD44, EGFR, E-cadherin, and vimentin can be used as potential predictive markers of recurrence as well as potential predictive markers of survival to identify aggressive lesions in cases of OSCC.

The new knowledge generated is the computed score for CD44, EGFR, E-cadherin, and vimentin, which can be used as indicators to predict tumor aggressiveness by implicating recurrence. With the help of these biomarkers' assessments, evidence was generated that patient survival can be predicted at the time of diagnosis, thereby helping the clinician to customize treatment protocol for each patient based on the expression of these markers. The limitations of the study were that a larger sample size could not be included due to the loss of patients on follow-up, and the sample included was unicentric.

## References

[1] Underhill C (1992). CD44: the hyaluronan receptor. J Cell Sci.

[2] Kaur G, Carnelio S, Rao N, Rao L (2009). Expression of E-cadherin in primary oral squamous cell carcinoma and metastatic lymph nodes: an immunohistochemical study. Indian J Dent Res.

[3] Salomon DS, Brandt R, Ciardiello F, Normanno N (1995). Epidermal growth factor-related peptides and their receptors in human malignancies. Crit Rev Oncol Hematol.

[4] Liu LK, Jiang XY, Zhou XX, Wang DM, Song XL, Jiang HB (2010). Upregulation of vimentin and aberrant expression of E-cadherin/beta-catenin complex in oral squamous cell carcinomas: correlation with the clinicopathological features and patient outcome. Mod Pathol.

[5] Krejcie RV, Morgan DW (1970). Determining sample size for research activities. Educ Psychol Meas.

[6] Hema K, Rao K, Devi HU, Priya N, Smitha T, Sheethal H (2014). Immunohistochemical study of CD44s expression in oral squamous cell carcinoma — its correlation with prognostic parameters. J Oral Maxillofac Pathol.

[7] Sarkis SA, Abdullah BH, Majeed BAA, Talabani NG (2010). Immunohistochemical expression of epidermal growth factor receptor (EGFR) in oral squamous cell carcinoma in relation to proliferation, apoptosis, angiogenesis and lymphangiogenesis. Head Neck Oncol.

[8] Doshi N, Shah S, Patel K, Jhabuawala M (2011). Histological grading of oral cancer: a comparision of different systems and their relation to lymph node metastasis. Natl J comm Med.

[9] Urist M, O'Brien CJ, Soong SJ, Visscher DW, Maddox WA (1987). Squamous cell carcinoma of the buccal mucosa: analysis of prognostic factors. Am J Surg.

[10] Liu CH, Chen HJ, Wang PC, Chen HS, Chang YL (2013). Patterns of recurrence and second primary tumors in oral squamous cell carcinoma treated with surgery alone. Kaohsiung J Med Sci.

[11] Kernohan MD, Clark JR, Gao K, Ebrahimi A, Milross CG (2010). Predicting the prognosis of oral squamous cell carcinoma after first recurrence. Arch Otolaryngol Head Neck Surg.

[12] Williams HK, Sanders DS, Jankowski JA, Landini G, Brown AM (1998). Expression of cadherins and catenins in oral epithelial dysplasia and squamous cell carcinoma. J Oral Pathol Med.

[13] Mehendiratta M, Solomon MC, Boaz K, Guddattu V, Mohindra A (2014). Clinico-pathological correlation of E-cadherin expression at the invasive tumor front of Indian oral squamous cell carcinomas: an immunohistochemical study. J Oral Maxillofac Pathol.

[14] Gulati N, Rathore AS, Juneja S, Rastogi P (2017). Expression of E-cadherin and B-cell lymphoma 2 in oral cancer: a ratio-based planning for targeted therapy. Indian J Dent Res.

[15] de Araujo VC, Pinto Júnior DS, de Sousa SO, Nunes FD, de Araujo NS (1993). Vimentin in oral squamous cell carcinoma. Eur Arch Otorhinolaryngol.

[16] Irani S, Dehghan A (2018). The expression and functional significance of vascular endothelial-cadherin, CD44, and vimentin in oral squamous cell carcinoma. J Int Soc Prev Community Dent.

[17] Ramaeker F, Puts J, Kant A, Moesker O, Jap P, Voojis P (1982). Differential diagnosis of human carcinomas, sarcomas and their metastases using antibodies to intermediate-sized filaments. Eur J Cancer Clin Oncol.

[18] Costa LC, Leite CF, Cardoso SV, Loyola AM, Faria PR, Souza PE, Horta MC (2015). Expression of epithelial-mesenchymal transition markers at the invasive front of oral squamous cell carcinoma. J Appl Oral Sci.

